# The multifaceted role of beta‐blockers in overcoming cancer progression and drug resistance: Extending beyond cardiovascular disorders

**DOI:** 10.1096/fj.202400725RR

**Published:** 2024-07-08

**Authors:** Simona Cavalu, Sameh Saber, Ahmed E. Amer, Rabab S. Hamad, Mustafa Ahmed Abdel‐Reheim, Elsayed A. Elmorsy, Amir Mohamed Abdelhamid

**Affiliations:** ^1^ Faculty of Medicine and Pharmacy University of Oradea Oradea Romania; ^2^ Department of Pharmacology, Faculty of Pharmacy Delta University for Science and Technology Gamasa Egypt; ^3^ Biological Sciences Department, College of Science King Faisal University Al Ahsa Saudi Arabia; ^4^ Central Laboratory Theodor Bilharz Research Institute Giza Egypt; ^5^ Department of Pharmaceutical Sciences, College of Pharmacy Shaqra University Shaqra Saudi Arabia; ^6^ Department of Pharmacology and Toxicology, Faculty of Pharmacy Beni‐Suef University Beni Suef Egypt; ^7^ Department of Pharmacology and Therapeutics, College of Medicine Qassim University Buraidah Saudi Arabia; ^8^ Department of Clinical Pharmacology, Faculty of Medicine Mansoura University Mansoura Egypt

**Keywords:** angiogenesis, apoptosis, beta‐blockers, cancer, drug resistance, metastasis

## Abstract

Beta‐blockers are commonly used medications that antagonize β‐adrenoceptors, reducing sympathetic nervous system activity. Emerging evidence suggests that beta‐blockers may also have anticancer effects and help overcome drug resistance in cancer treatment. This review summarizes the contribution of different isoforms of beta‐adrenoceptors in cancer progression, the current preclinical and clinical data on associations between beta‐blockers use and cancer outcomes, as well as their ability to enhance responses to chemotherapy and other standard therapies. We discuss proposed mechanisms, including effects on angiogenesis, metastasis, cancer stem cells, and apoptotic pathways. Overall, results from epidemiological studies and small clinical trials largely indicate the beneficial effects of beta‐blockers on cancer progression and drug resistance. However, larger randomized controlled trials are needed to firmly establish their clinical efficacy and optimal utilization as adjuvant agents in cancer therapy.

## Graphical abstract

*Modulation of the Tumor Microenvironment by β‐Blockers*: The tumor microenvironment is critical for cancer progression and therapeutic resistance. Cancer‐associated fibroblasts and epithelial‐to‐mesenchymal transition promote a supportive tumor stroma and invasion. Angiogenesis, mediated by vascular endothelial growth factor, allows nutrient and oxygen delivery. Meanwhile, cytokine signaling enables immune evasion. Beta‐blockers may reshape the tumor microenvironment by inhibiting angiogenesis, modifying immune cell infiltration and function, disrupting catecholaminergic niche signaling, and attenuating surgery‐induced immunosuppression. These effects highlight the potential of beta‐blockers to modulate the tumor microenvironment as a novel strategy to improve cancer therapy.

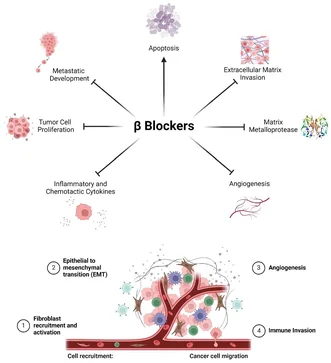

**Abbreviations**
5‐FU: 5‐fluorouracil
Akt: protein kinase B
AR: adrenoceptor
BCL‐2: B‐cell lymphoma 2
CDDP: cisplatin
COX‐2: cyclooxygenase 2
EPAC: exchange protein activated by adenylyl cyclase
ERK: extracellular signal–regulated kinase
MEK: mitogen‐activated protein kinase
MMP: matrix metalloproteinase
NF‐kB: nuclear factor kappa‐light‐chain‐enhancer of activated B cells
PAP: phosphatidate phosphatase
PD‐1: programmed cell death protein 1
PD‐L1: programmed cell death ligand 1
PD‐L1: programmed death ligand 1
p‐PKA: phospho‐protein kinase A
PTEN: phosphatase, and tensin homolog
Rap1A: Ras‐like guanine triphosphatase.
S1P2: S1P receptor 2
SK2: sphingosine kinase 2
TKI: tyrosine kinase inhibitor
TNBC: triple‐negative breast cancer
VEGF: vascular endothelial growth factor

## 1 INTRODUCTION

Beta‐blockers, also known as beta‐adrenergic blocking agents, are a class of medications that antagonize β‐adrenoceptors, blocking the binding and effects of norepinephrine and epinephrine. This results in reduced blood pressure and heart rate via decreased cardiac output.^1^ Beta‐blockers such as propranolol are commonly prescribed for conditions including hypertension, coronary artery disease, arrhythmias, migraines, and anxiety.^2^

Propranolol is a non‐selective beta‐adrenoceptor antagonist/inverse agonist that has been widely studied in the context of cancer progression and resistance. While initially developed as a cardiovascular agent, propranolol has been shown to exhibit additional properties relevant to its potential anticancer effects. Notably, it has been demonstrated to activate ERK phosphorylation, a signaling pathway implicated in various cellular processes, including proliferation and survival.^3^ Furthermore, studies identified what appeared to be an off‐target effect of propranolol, attributed to its membrane‐stabilizing property.^4^, ^5^

Carvedilol, another beta‐blocker frequently investigated in cancer research, exhibits distinct pharmacological characteristics. It is a non‐selective beta‐adrenoceptor antagonist with additional alpha‐1 adrenoceptor blocking activity.^6^ Importantly, carvedilol has been identified as a biased ligand, preferentially activating the β‐arrestin signaling pathway over the canonical G protein‐mediated pathway.^3^, ^7^, ^8^ This unique property of carvedilol may contribute to its specific effects on cellular processes relevant to cancer progression and resistance.

Nadolol, a non‐selective beta‐adrenoceptor antagonist without intrinsic sympathomimetic activity, has been studied in various cancer contexts. While it exhibits traditional beta‐blocking properties, its specific mechanisms of action in cancer remain to be fully elucidated.^9^, ^10^

Atenolol and bisoprolol, which are selective beta‐1 adrenoceptor antagonists, have also been investigated for their potential anticancer effects. Its selectivity for the beta‐1 subtype may contribute to differential effects on cellular processes compared to non‐selective beta‐blockers.^11^, ^12^

By understanding the distinct pharmacological profiles of these beta‐blockers, researchers can gain insights into their potential mechanisms of action and therapeutic effects in cancer. The varying selectivity‐biased signaling properties and additional characteristics of these compounds may contribute to their differential impacts on cellular processes underlying cancer progression and resistance. In recent years, there has been growing interest in the potential anticancer effects of beta‐blockers. Both preclinical and epidemiological studies have suggested that beta‐blockers may inhibit cancer progression and improve outcomes in certain malignancies, such as breast, ovarian, and prostate cancers.^13^, ^14^, ^15^, ^16^, ^17^, ^18^, ^19^ In addition, beta‐blockers may help sensitize cancer cells to chemotherapy and other standard therapies, reversing drug resistance, which poses a major obstacle to cancer treatment.^12^, ^16^, ^20^

While some beta‐blockers like propranolol exhibit anticancer properties, others like bisoprolol do not. Some of them are biased ligands and can activate the beta‐arrestin‐ERK pathway, some are weak agonists, and some are inverse agonists. The anticancer effects of beta‐blockers may depend on the specific type of beta‐blocker, and the underlying mechanisms are not fully elucidated. Further research is needed to clarify the potential role of beta‐blockers in cancer prevention and treatment. This review summarizes current evidence from preclinical and clinical studies regarding the effects of beta‐blockers on cancer progression, drug resistance, radiotherapy, and immunotherapy. We also discuss proposed biological mechanisms that could mediate the observed anticancer and chemo‐sensitizing effects of beta‐blockers.

## 2 IMPACT OF THE DIFFERENT SUBTYPES OF β‐ADRENOCEPTORS ON CANCER PROGRESSION

The β‐adrenoceptor consists of three subtypes, β1, β2, and β3, which are found in various sites associated with tumor growth and metastasis, such as the brain, lung, liver, kidney, adrenal gland, breast, ovary, prostate, lymphoid tissues, bone marrow, and vasculature.^9^ β‐adrenergic signaling plays a crucial role in regulating the biological activity of different cell types relevant to cancer, including epithelial cells, vascular myocytes and pericytes, adipocytes, fibroblasts, neural/glial cells, as well as lymphoid and myeloid immune cells.^21^, ^22^

The beta‐2 adrenoceptor (β2‐AR) exhibits a remarkable ability to couple to multiple signaling pathways, a phenomenon known as pleiotropic coupling. While the canonical pathway associated with β2‐AR activation involves the stimulation of the Gs protein, leading to an increase in cyclic AMP (cAMP) levels and subsequent activation of protein kinase A (PKA), Emerging evidence demonstrates that the β2‐AR can activate unique signaling pathways. Current research suggests that β2‐AR signaling is modulated through interactions with various proteins, including guanosine triphosphate‐binding proteins like Gs and Gi, protein kinases such as cAMP‐dependent protein kinase, protein kinase C, G‐protein‐coupled receptor kinases, and tyrosine kinases, as well as adaptor proteins like arrestins, A‐kinase anchoring proteins, and the Na^+^/H^+^‐exchanger regulatory factor.^23^

Notably, the β2‐AR has been shown to engage the β‐arrestin pathway, which can lead to the activation of various downstream signaling events, including the mitogen‐activated protein kinase (MAPK) cascade. This pathway has been implicated in cellular processes such as proliferation, survival, and migration, which are crucial in the context of cancer progression and metastasis.^24^, ^25^, ^26^, ^27^, ^28^

The pleiotropic coupling abilities of the β2‐AR have significant implications in the context of cancer progression and resistance. Different signaling pathways may be differentially involved in regulating cellular processes such as proliferation, apoptosis, and metastasis.^29^ Consequently, the ability of the β2‐AR to engage multiple signaling cascades may contribute to its complex and multifaceted roles in various aspects of cancer development and treatment resistance. β2‐ARs, which are widely expressed in prostate luminal cells and are receptive to sympathetic impulses, are important in regulating numerous aspects of prostate cancer cell activity. They have been shown in studies to be involved in the transdifferentiation of cancer cells into neuroendocrine‐like cells. They also have an impact on processes such as apoptosis, angiogenesis, epithelial‐mesenchymal transition, cell migration, and metastasis.^30^ Also, they were shown to be the most dominant in tumor‐related pathways until a few years ago.^31^

However, growing evidence suggests that β3‐adrenoceptors play a role in the antioxidant defense system in cancer. These receptors were found to be implicated in tumor proliferation, angiogenesis, metastasis, and immune tolerance. Moreover, inhibition of β3‐adrenoceptors using the preferential antagonist SR59230A has been shown to increase reactive oxygen species (ROS) levels in cancer cells with subsequent cell death, decrease nitric oxide (NO) levels, and inhibit angiogenesis.^32^ Recently, blocking β3‐adrenoceptors has been found to promote the reactivation of the immune response, partly through modulation of the programmed cell death Protein 1 (PD‐1)/programmed cell death Ligand 1 (PD‐L1) signaling axis. Notably, the blockade of β3‐adrenoceptors in tumor‐infiltrating lymphocytes diminished their ability to secrete interferon‐γ, resulting in reduced PD‐L1 expression on neuroblastoma tumor cells, which is influenced by TILs infiltration. Furthermore, the high expression of the β3‐adrenoceptor gene in neuroblastomas was shown to be associated with worse clinical outcomes compared to the low expression group.^33^ The β3‐adrenoceptor has been shown to play a key role in the regulation of proliferation and the balance between stemness and differentiation in neuroblastoma through crosstalk with the sphingosine kinase 2 (SK2)/S1P receptor 2 (S1P2) axis.^31^ The growth and progression of neuroblastoma were shown to be suppressed by the specific antagonism of β3‐adrenoceptors using SR59230A.^31^ Another study showed that the administration of the preferential β1‐adrenoceptor antagonist, atenolol, resulted in a modest reduction in tumor growth without any effect on the development of mechanical allodynia in mice bearing K7M2 osteosarcoma. This study proposed that the antagonism of β2‐ and β3‐adrenoreceptors contributes not only to the reduction of tumor growth but also to the alleviation of cancer‐induced pain.^34^

## 3 PRECLINICAL STUDIES

Numerous preclinical studies have demonstrated the anticancer effects of beta‐blockers in cell lines and animal models across a range of cancer types (Table 1). In vitro experiments have demonstrated that norepinephrine possesses a strong capacity to stimulate migratory activity in cell lines associated with colon, prostate, ovarian, and breast cancer. Conversely, the inhibition of β‐adrenoceptors appears to have a positive impact on the immune response and the regulation of cytokines.^9^, ^46^, ^47^, ^48^ In breast cancer models, beta‐blockers including propranolol reduced tumor growth and metastasis.^11^, ^49^, ^50^ Propranolol also inhibited ovarian and prostate cancer growth in rodent models.^39^, ^51^, ^52^ Furthermore, propranolol was reported to block the late phase of autophagy induced by 2‐deoxyglucose as a pro‐survival mechanism and therefore promote prostate cancer cell death through inhibition of autophagic flux and accumulation of autophagic vesicles, which consequently inhibits cell proliferation and induces cellular apoptosis.^39^ The ability of propranolol to inhibit autophagic flux is attributed to its known suppression activity on the phosphatidate phosphatase (PAP) of lipins.^53^

**TABLE 1:** Summary of key preclinical studies on beta‐blockers in cancer models.

Cancer type	Beta‐blocker used	Key findings	Proposed mechanisms	Reference
Breast	Propranolol	Inhibited proliferation, migration, and invasion of breast cancer cells	Suppression of phosphorylation of p38 MAPK in	[35]
Breast	Propranolol	Suppressed the viability of human breast adenocarcinoma, hindered the progression of the cell cycle at G0/G1 and S phase, and induced cellular apoptosis	Diminished the phosphorylation of (ERK)1/2 and the cyclo‐oxygenase 2 (COX‐2) expression	[36]
Breast	Carvedilol	Blocking of neural regulation of breast cancer progression, reducing primary tumor growth and metastasis	Blocking sympathetic nervous system activation and β‐adrenoceptor signaling	[37]
Skin	Carvedilol	Prevented UV‐induced skin tumor development	Inhibition of UV‐induced transcriptional activity of NF‐κB and Activator protein 1 (AP‐1)	[38]
Prostate	Propranolol +2DG	Synergistic anti‐tumor effects	Autophagy blocking through the suppression activity on phosphatidate phosphatase (PAP) of lipins	[39]
Ovarian	Propranolol	Reduced metastasis	Downregulation of MMPs	[40]
Ovarian	Propranolol	Induced apoptosis	Reduced expression of the anti‐apoptotic protein BCL‐2	[41]
Prostate and bladder	Propranolol	Increased apoptosis	Activation of caspase‐3 and encouraging the expression of BAX, Wee1, GADD153, and apoptosis‐inducing Suppression of BCL‐2, ERK, and JNK	[42]
Sarcoma	Propranolol	Chemo‐sensitization, reduced viability	Modulation of drug efflux, apoptosis	[43]
Colorectal	Propranolol	Disrupted hypoxic adaptation, chemo‐sensitization	Effects on HIF‐1α, pH regulation	[44]
Oral	Propranolol	Reduced viability, migration	Effects on NF‐kB, VEGF, Akt/PTEN	[45]

Several studies have elucidated the potential mechanisms underlying the anticancer effects of beta‐blockers observed in preclinical models. Beta‐blockers have been found to inhibit angiogenesis, or the formation of new blood vessels, in cell and animal models of ovarian, prostate, and oral cancers.^54^, ^55^, ^56^ Moreover, a recent study demonstrated the apoptotic capability of propranolol in prostate and bladder cancer cell lines through activation of caspase‐3 and encouraging the expression of BAX, Wee1, GADD153, and apoptosis‐inducing factor. Moreover, propranolol was found to suppress BCL‐2 expression in addition to JNK and ERK activities in the same study.^42^

In breast cancer models, propranolol reduced the expression of vascular endothelial growth factor (VEGF), a key pro‐angiogenic factor.^57^ Beta‐blockers also decreased cancer cell proliferation and survival in some preclinical studies.^58^

Propranolol markedly suppressed the viability of the human breast adenocarcinoma cell line MDA‐MB‐231 cells, hindered the progression of the cell cycle at G0/G1 and S phases, and induced cellular apoptosis. Furthermore, propranolol significantly diminished the phosphorylation of (ERK)1/2 and the cyclo‐oxygenase 2 (COX‐2) expression.^36^ Furthermore, MCF‐7 and MDA‐MB‐231 breast cancer cells were found to express beta‐adrenoceptors. It was observed that epinephrine stimulated the proliferation, migration, and invasion of breast cancer cells. Epinephrine treatment also increased the phosphorylation of p38 MAPK in the cells and promoted tumor growth in vivo. However, these effects were significantly reduced by the administration of propranolol.^35^

Furthermore, beta‐blockers have been shown to inhibit metastasis or the spread of cancer to distant sites. In an orthotopic model of ovarian cancer, propranolol significantly reduced metastasis compared to control.^16^, ^20^, ^55^ Downregulation of matrix metalloproteinases (MMPs), proteins involved in extracellular matrix breakdown and cell invasion, was proposed as a potential mechanism for the antimetastatic effects.^40^, ^59^, ^60^ Moreover, in human ovarian cancer cells, propranolol induced apoptosis and reduced expression of the anti‐apoptotic protein BCL‐2.^41^ Taken together, preclinical data indicate beta‐blockers can inhibit cancer progression through multiple mechanisms, providing biological plausibility for translational studies in human cancers.

A growing number of preclinical studies have demonstrated that beta‐blockers can re‐sensitize resistant cancer cells and enhance their responses to chemotherapy drugs both in vitro and in vivo. Preclinical studies have provided evidence that the beta‐blocker propranolol can help sensitize drug‐resistant cancer cells and enhance responses to chemotherapy. In sarcoma models, propranolol synergized with doxorubicin or docetaxel to inhibit the viability of diverse sarcoma subtypes, including angiosarcoma, leiomyosarcoma, and liposarcoma. The chemo‐sensitizing effects occurred with both beta‐blocker receptor‐inhibiting and non‐inhibiting enantiomers of propranolol, indicating β‐adrenoceptor‐independent mechanisms.^61^ Mechanistically, propranolol increased cytoplasmic doxorubicin concentrations in sarcoma cells by reducing lysosomal sequestration and efflux of the drug. It also decreased the activity of the multidrug resistance efflux pump P‐glycoprotein, thereby increasing intracellular doxorubicin levels. Furthermore, propranolol attenuated Akt‐dependent pro‐survival signaling, and NF‐κB/COX‐2 pathway activation induced by doxorubicin and docetaxel, respectively, enhancing cell sensitivity.^43^

Recent evidence indicates the non‐selective beta‐blocker propranolol may act as a chemo‐sensitizing agent in colorectal cancer models, including in 5‐fluorouracil‐resistant cells. Propranolol was found to disrupt the hypoxic adaptation of cancer cells by inhibiting HIF‐1α and carbonic anhydrase IX, key regulators of the tumor microenvironment. This led to the activation of apoptotic pathways and reduced growth of colorectal cancer cell xenografts, even in cells adapted to propranolol. The study highlights propranolol's ability to modulate signaling pathways that control the tumor microenvironment and influence the chemotherapeutic response. Blockade of β‐adrenoceptors was shown to disrupt microenvironment homeostasis and sensitize colorectal cancer cells to chemotherapy.^44^

Overall, these preclinical findings provide evidence that repurposing propranolol as a chemosensitizer in combinatorial regimens could help overcome drug resistance mediated by the tumor microenvironment. A recent study found that the beta‐blocker propranolol reduced the viability of oral squamous cell carcinoma cells and enhanced the antitumor effects of cisplatin (CDDP) and 5‐fluorouracil (5‐FU).^45^ Propranolol dose‐dependently eliminated oral cancer cells and significantly augmented the cytotoxicity of CDDP and 5‐FU.^45^ Propranolol was shown to modulate multiple signaling pathways implicated in tumor progression and drug resistance, including downregulation of NF‐κB and VEGF and effects on the Akt/PTEN pathway.^45^, ^62^ Notably, the role of NF‐κB in inflammation and cancer progression has been extensively studied.^63^, ^64^, ^65^, ^66^, ^67^, ^68^ Propranolol also inhibits oral cancer cell migration.^45^ Overall, this preclinical study provides evidence that propranolol can sensitize oral squamous cell carcinomas to chemotherapy through a variety of mechanisms. The chemo‐sensitizing and anti‐migratory effects further support the potential utility of propranolol as an adjunct to standard chemotherapy regimens in oral cancer. On the other hand, a recent study found that carvedilol blocks the neural regulation of breast cancer progression, reducing primary tumor growth and metastasis in a mouse model of breast cancer.^37^ In the same study, carvedilol treatment effectively blocked the isoprenaline‐induced invasion of human breast cancer cell lines through an extracellular matrix. This study proposed that carvedilol's anticancer effects in breast cancer involve blocking sympathetic nervous system activation and β‐adrenoceptor signaling, ultimately slowing cancer progression.^37^ Moreover, carvedilol prevented UV‐induced skin tumor development by inhibiting UV‐induced transcriptional activity of NF‐κB and activator protein 1 (AP‐1).^38^ In contrast, bisoprolol, a selective β1‐adrenoceptor blocker, did not demonstrate any cytotoxic effects or impact on cell proliferation in breast cancer cell lines.^69^

## 4 CLINICAL AND EPIDEMIOLOGICAL STUDIES

Several retrospective epidemiological studies have evaluated associations between beta‐blocker use and cancer outcomes in various malignancies (Table 2). The majority of research has focused on common cancers such as breast, prostate, and ovarian cancers. In many types of cancer, multiple studies found beta‐blocker use was associated with reduced cancer‐specific and overall mortality.^74^, ^75^, ^76^, ^77^ A meta‐analysis found that beta‐blocker use was associated with significantly reduced breast cancer mortality.^78^ The associations appear strongest for early‐stage or localized diseases.^77^ Proposed mechanisms include inhibition of angiogenesis and metastasis by blocking stress‐induced signals.

**TABLE 2:** Summary of key clinical studies on beta‐blockers and cancer outcomes.

Cancer type	Study design	Sample size	Key findings	Reference
Breast	Retrospective cohort	>30 000	No overall survival benefit, benefit only in the TNBC subtype	[15]
Breast	Retrospective analysis	>4000	Reduced breast cancer‐specific mortality	[37]
Prostate	Meta‐analysis	~17 000	Reduced cancer‐specific mortality, no effect on overall mortality	[70]
Prostate	Retrospective cohort	>11 000	Lower recurrence with perioperative beta‐blocker use	[71]
Ovarian	Retrospective cohort	>1400	Improved overall survival with non‐selective beta‐blockers	[72]
Ovarian	Population‐based cohort	>9000	Increased mortality associated with beta‐blocker use	[12]
Lung	Retrospective analyses	Varied	Mixed results need prospective trials	[73]

The potential anticancer effects of beta‐blockers in prostate cancer have been examined in multiple clinical studies, with conflicting results on their ability to improve outcomes. A cohort study of over 3500 prostate cancer patients with high‐risk or metastatic disease at diagnosis found that beta‐blocker use was associated with reduced prostate cancer‐specific mortality.^79^ This suggests beta‐blockers may improve survival, specifically from prostate cancer, in patients with advanced disease. However, a meta‐analysis of nearly 17 000 prostate cancer patients observed reduced cancer‐specific mortality with beta‐blockers but no effect on all‐cause mortality.^70^ The lack of association with all‐cause mortality tempers the evidence that beta‐blockers confer a survival advantage. Meanwhile, an analysis of over 11 000 prostate cancer patients undergoing radical prostatectomy found that perioperative beta‐blocker use significantly correlated with lower rates of treatment for cancer recurrence.^71^ This indicates beta‐blockers may help prevent disease relapse after definitive treatment.

The evidence for beta‐blockers improving clinical outcomes in ovarian cancer has been mixed. A multicenter review of over 1400 ovarian cancer patients found that the use of non‐selective beta‐blockers was associated with longer overall survival.^72^ This suggests non‐selective beta‐blockers may confer a survival advantage. However, a larger population‐based study of over 9000 ovarian cancer patients found that beta‐blocker use, both non‐selective and cardio‐selective types, was associated with increased mortality.^12^, ^80^ The decreased survival among beta‐blocker users may be explained by the greater prevalence of cardiovascular disease and comorbidities in older patients more likely to be prescribed beta‐blockers. A limitation was the lack of data on potential confounders like comorbidities that could impact survival.^12^, ^80^ The conflicting results from these two studies underscore the complexity of assessing beta‐blocker effects on ovarian cancer outcomes. While the first study found improved survival with non‐selective beta‐blocker use, the second, larger study associated beta‐blockers with increased mortality, possibly due to comorbidities in beta‐blocker users. Additional research is required to account for confounding factors to clarify the effects of different beta‐blocker types on ovarian cancer prognosis.

Clinical data evaluating beta‐blocker effects on outcomes in breast and lung cancer has shown mixed results. A large cohort study of over 30 000 breast cancer patients found no association between beta‐blocker use and breast cancer‐specific survival overall.^15^ However, improved survival was seen specifically in patients with triple‐negative breast cancer (TNBC). This selective benefit in TNBC was confirmed in a meta‐analysis showing beta‐blockers were associated with better progression/recurrence‐free and breast cancer‐specific survival only in this subtype.^15^ Meanwhile, a study in patients with EGFR‐mutant lung adenocarcinoma found former beta‐blocker use prolonged survival in those receiving first‐line EGFR tyrosine kinase inhibitors (TKIs).^73^ Furthermore, a retrospective analysis of a large cohort of patients with breast cancer revealed that women using carvedilol at breast cancer diagnosis had reduced breast cancer‐specific mortality compared to those who did not. The 5‐year cumulative incidence of breast cancer deaths was 3.1% in carvedilol users versus 5.7% in non‐users.^37^

Taken together, these studies provide signals that beta‐blockers may improve outcomes for certain molecular subtypes of breast cancer and augment the efficacy of targeted therapies like EGFR‐TKIs in lung cancer. However, the retrospective analyses are subject to limitations, such as potential residual confounding. Large prospective trials are required to firmly establish if incorporating beta‐blockers into treatment regimens benefits specific patient subsets across cancer types. Elucidating optimal beta‐blocker combinations with standard therapies will also be key in future research. Furthermore, prospective randomized trials are imperative to establish if incorporating beta‐blockers into treatment regimens can enhance clinical outcomes in this patient population. While epidemiological data largely supports the anticancer effects of beta‐blockers, confounding factors may impact associations in retrospective studies. Confounders such as comorbidities and socioeconomic status, which influence both beta‐blocker use and cancer outcomes, are difficult to fully account for. Therefore, randomized controlled trials are critical to establish definitive clinical evidence.

## 5 IMPACT OF BETA‐BLOCKERS ON CURRENT TREATMENTS

Propranolol was shown to sensitize human gastric adenocarcinoma cell lines (SGC‐7901 and BGC‐823) to radiation therapy through suppression of the downstream NF‐κB‐VEGF/EGFR/COX‐2 pathway.^81^ Moreover, beta‐blockers have been found to markedly improve the radiotherapy efficacy in medulloblastoma by disrupting adaptive bioenergetic capacities, which consequently cause the overproduction of superoxide radicals and eventually enhance ionizing radiation‐intermediated DNA damages.^82^ Likewise, in vitro treatment of adenocarcinoma cell lines (PC9 and A549) with propranolol combined with radiation or cisplatin diminished clonogenic survival of the lung. It suppressed the expression of phospho‐protein kinase A (p‐PKA) in both cell lines relative to radiotherapy or cisplatin alone.^83^ A retrospective cohort study of 722 non‐small‐cell lung cancer patients revealed that beta‐blockers improved outcomes, with markedly better distant metastasis‐free survival and overall survival in those patients who received definitive radiotherapy.^84^ Another retrospective cohort study demonstrated that b‐blockers show earlier and maintained outcomes in low‐ to medium‐grade intracranial meningiomas that were stereotactic irradiated.^85^ Recently, a retrospective cohort study of 291 esophageal cancer patients who received chemoradiation reported that concurrent use of beta‐blockers was associated with enhancing progression‐free survival and overall survival.^86^ On the other hand, in a study using a mouse model of melanoma, the combination of propranolol and IL‐2 or αPD‐1 immunotherapies significantly delayed tumor growth relative to immunomonotherapy. Furthermore, the same study performed a retrospective analysis of 195 metastatic melanoma patients, which demonstrated an improvement in overall survival in patients treated with beta‐blockers as an adjuvant to immunotherapy compared to immunotherapy alone.^87^ In another retrospective study of 109 non‐small cell lung cancer patients, progression‐free survival was attenuated among patients treated with beta‐blockers and immune checkpoint inhibitors relative to immune checkpoint inhibitors without beta blockers.^88^ Most recently, a phase III clinical trial of stage IIIA, IIIB, or IIIC melanoma patients suggested that beta‐blockers may improve the efficiency of adjunct pembrolizumab treatment.^89^

## 6 MECHANISMS OF ACTION

Catecholamines like epinephrine and norepinephrine are secreted in response to stress and can promote cancer progression through multiple mechanisms.^9^ Many tumors express adrenoceptors that allow them to respond to catecholamine signals.^90^ Epidemiological studies have associated major life stressors with increased tumor progression and poorer cancer outcomes. Subsequent preclinical research found catecholamines enhance metastasis and suppress anti‐tumor immunity. Catecholamine signaling through β‐adrenoceptors activates intracellular pathways involved in cancer promotion, including cAMP‐PKA.^91^ Overall, evidence indicates that stress‐induced catecholamine release can fuel tumor growth and metastasis through adrenergic signaling. This represents pathways by which psychological stress may adversely impact cancer outcomes.

Adrenoceptors (ARs) belong to a group of G‐protein‐coupled transmembrane receptors (GPCRs) that facilitate cellular growth and viability^92^ upon interaction with small ligands like catecholamines, specifically adrenaline and noradrenaline.^93^ Excessive stimulation or disruptions in GPCR signaling pathways often contribute to the development of cancer. This is due to the disruption of critical processes, including cell survival and proliferation, evasion of the immune system, promotion of tumor blood vessel formation (angiogenesis), invasion into surrounding tissues, metastasis, and resistance to therapeutic drugs.^94^ When norepinephrine (NE) and epinephrine (E) bind to β‐receptors, they activate the Gαs guanine nucleotide‐binding protein, leading to the stimulation of adenylyl cyclase and the production of cyclic 3′–5′ adenosine monophosphate (cAMP). This transient increase in cAMP levels can modulate a wide range of cellular processes through two major downstream effector systems.^9^ First, cAMP activates protein kinase A (PKA), which controls several cellular processes, including metabolism, growth, differentiation, neurotransmission, morphology, and proliferation.^95^ Moreover, cAMP activates the guanine nucleotide Exchange Protein activated by Adenylyl Cyclase (EPAC). EPAC induces B‐Raf, MEK1/2, and ERK1/2 through the activation of Ras‐like guanine triphosphatase (Rap1A). While the influence of β‐adrenergic on inflammation, invasion, and angiogenesis is controlled mainly by the PKA‐mediated induction of cytokines and growth factors encoding genes, EPAC governs the effects on cell morphology and motility.^9^ On the other hand, whereas β1‐AR and β2‐AR are primarily coupled to the stimulatory Gαs protein, the coupling of β3‐AR is more complex and can involve both Gαs and inhibitory Gαi proteins, depending on the tissue and species.^96^, ^97^ β3‐AR can also activate alternative signaling pathways, such as nitric oxide synthase and guanylyl cyclase, leading to increased cGMP levels.^96^

## 7 EFFECT OF BETA‐BLOCKERS ON MECHANISMS OF CANCER PROGRESSION

Beta‐blockers are proposed to modulate several mechanisms involved in cancer progression and drug resistance. Elucidating these molecular mechanisms is key to understanding how to maximize their clinical benefits (Figure 1).

**FIGURE 1:**
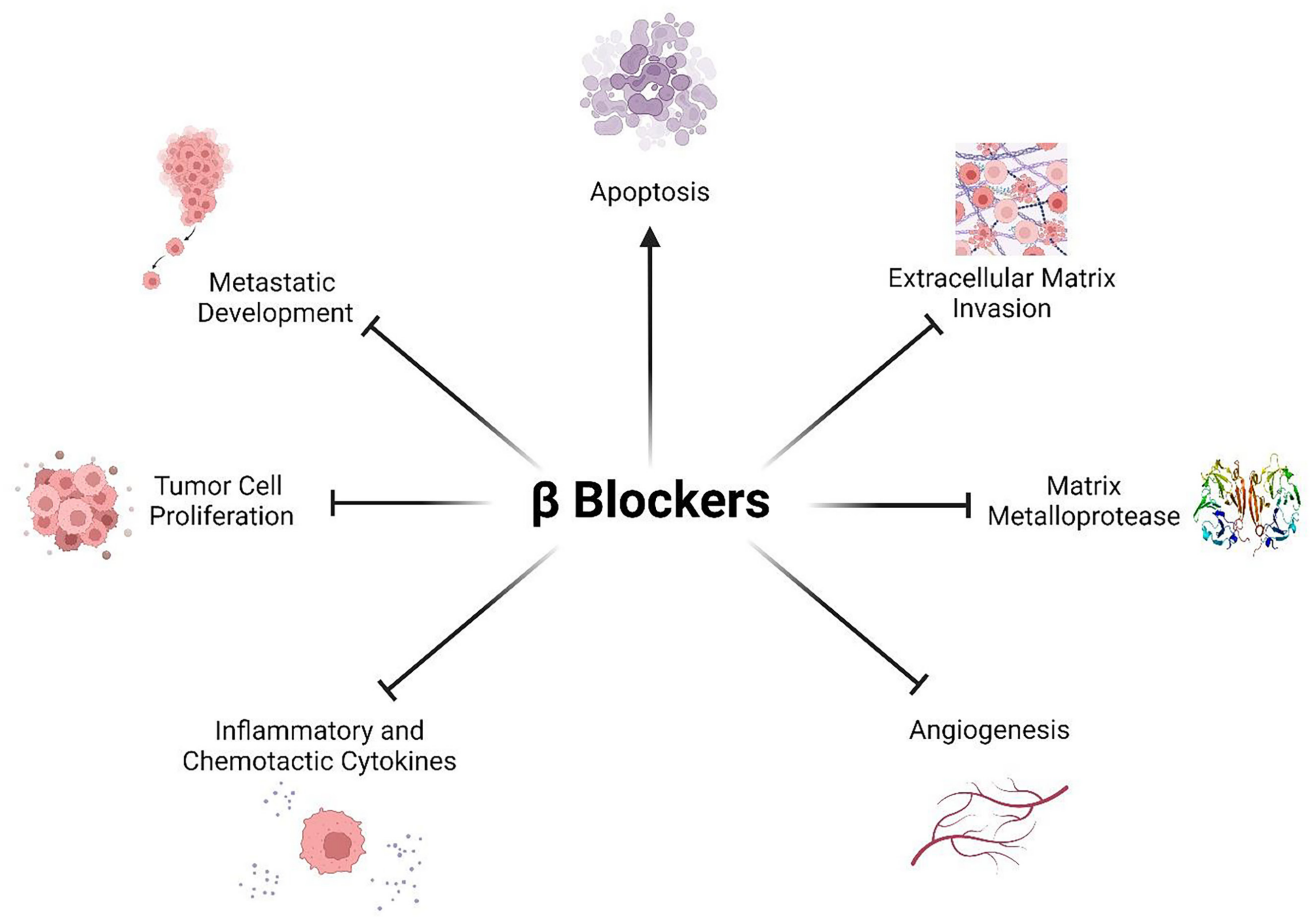
Proposed mechanisms of beta‐blockers in cancer treatment.

In the context of cancer progression, beta‐blockers have demonstrated their efficacy in hindering several critical aspects, including angiogenesis, metastasis, and the proliferation and survival of cancer cells,^55^ as previously elucidated. These effects have been attributed to the modulation of multiple signaling pathways. Preclinical investigations have indicated that the inhibition of β‐adrenoceptor (β‐AR) signaling through the use of a beta‐blocker can effectively impede various cellular mechanisms associated with the advancement of breast cancer. These mechanisms encompass the suppression of tumor cell proliferation, hindrance of metastatic development, induction of apoptosis, prevention of extracellular matrix invasion, suppression of matrix metalloprotease activation, reduction in the expression of inflammatory and chemotactic cytokines, inhibition of angiogenesis, and modulation of tumor immune responses.^14^, ^98^ Moreover, epidemiological and clinical data corroborate the potential translation of these anticancer properties into meaningful outcomes for human malignancies.

The intricate tumor microenvironment serves as a significant contributor to cancer progression and resistance to therapeutic interventions (Figure 2).^99^, ^100^ Emerging evidence suggests beta‐blockers may provide a novel approach to reshaping the tumor microenvironment and enhancing cancer treatment efficacy. The non‐selective beta‐blocker propranolol, used for cardiovascular conditions, has shown anti‐tumor effects in models of soft tissue sarcoma.^16^ Oral propranolol administration delayed tumor progression and increased survival by reducing angiogenesis and increasing T‐cell infiltration.^16^ It also upregulated PD‐L1 in tumor‐associated macrophages and improved immune checkpoint blockade outcomes.^16^ These results demonstrate propranolol's ability to modulate the immunosuppressive microenvironment.

**FIGURE 2:**
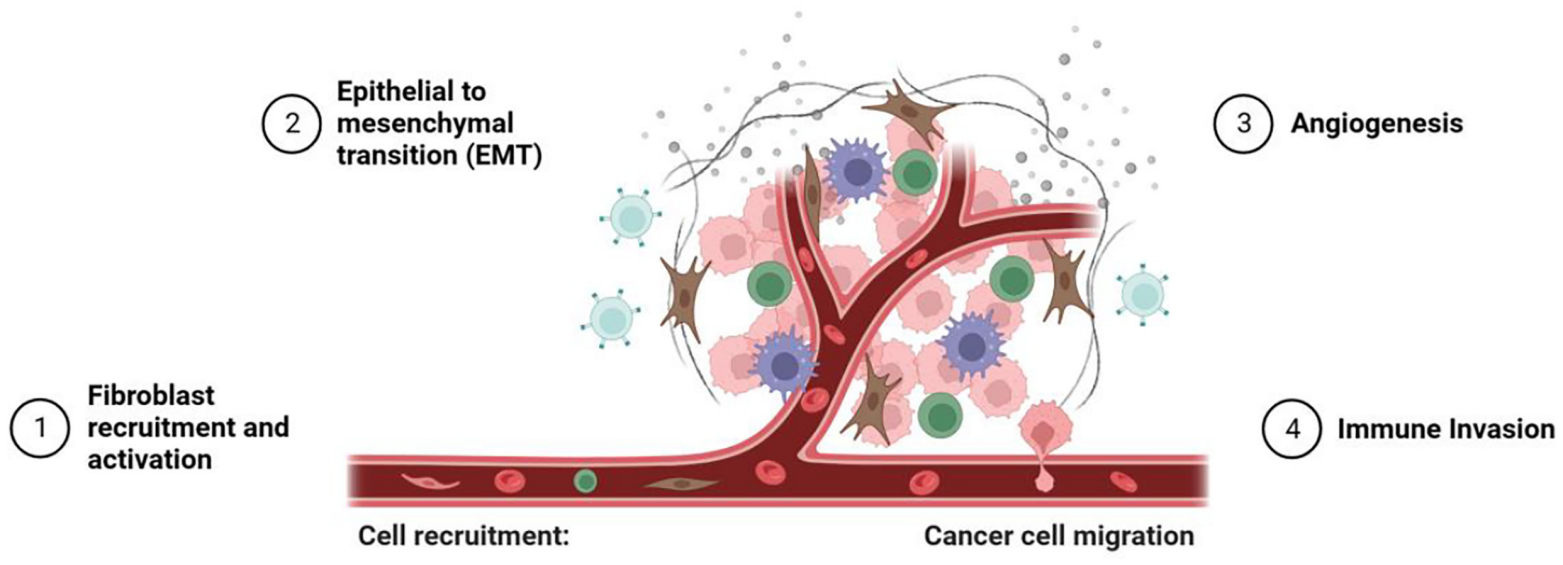
Modulation of the tumor microenvironment by beta‐blockers: The tumor microenvironment is critical for cancer progression and therapeutic resistance. Cancer‐associated fibroblasts and epithelial‐to‐mesenchymal transition promote a supportive tumor stroma and invasion. Angiogenesis, mediated by vascular endothelial growth factor, allows nutrient and oxygen delivery. Meanwhile, cytokine signaling enables immune evasion. Beta‐blockers may reshape the tumor microenvironment by inhibiting angiogenesis, modifying immune cell infiltration and function, disrupting catecholaminergic niche signaling, and attenuating surgery‐induced immunosuppression. These effects highlight the potential of beta‐blockers to modulate the tumor microenvironment as a novel strategy to improve cancer therapy.

Beta‐blockers may also target adrenergic signaling pathways in the innervated niche around tumors.^101^ Catecholamine‐mediated β‐adrenoceptor activation promotes proliferation, invasion, metastasis, and angiogenesis.^9^, ^102^ beta‐blockers could therefore disrupt this pro‐tumoral signaling. They may also improve immune surveillance by attenuating surgery‐induced immune suppression.^101^ Additionally, beta‐blockers reduce myeloid‐derived suppressor cells that mediate immunosuppression and limit immunotherapy efficacy.^101^ They may also promote cytotoxic CD8+ T cell activity.^103^ In summary, by reshaping the microenvironment through effects on angiogenesis, immune cell infiltration and activity, and innervated niche signaling, beta‐blockers may offer a novel pharmacological approach to improve cancer treatment.

While VEGF inhibitors reduce excessive angiogenesis in cancer,^59^, ^60^, ^104^, ^105^ they are associated with frequently inducing hypertension and arterial events as side effects.^106^, ^107^ The non‐selective beta‐blocker carvedilol may help reverse VEGF inhibitor‐induced hypertension through its vasodilatory properties.^106^, ^107^ In contrast, selective β1 or β2 blockers may promote VEGF‐mediated angiogenesis, unlike non‐selective agents like carvedilol.^101^

However, some clinical trials have reported increased mortality and recurrence with beta‐blocker use, though the mechanisms remain unclear.^108^, ^109^ The category of beta‐blockers appears to be important, as different agents may have distinct effects on VEGF signaling and angiogenesis. Careful attention to the risks and benefits of specific beta‐blocker types is therefore essential when investigating their utility in combination with VEGF inhibitors.

In the realm of drug resistance, a subset of tumor cells known as cancer stem cells has emerged as pivotal players due to their self‐renewal capabilities and their role in re‐establishing tumor diversity following chemotherapy.^110^ Preclinical studies have shown adrenergic signaling can interact with cancer stem cell properties to promote drug resistance. In lung cancer models, epinephrine treatment increased the cancer stem cell fraction in vitro, while stress reduction diminished stem cell marker expression and tumor growth in vivo.^111^ As cancer stem cells mediate resistance through their self‐renewal and tumor repopulation capacity, targeting their adrenergic regulation represents a potential approach for beta‐blockers to overcome resistance.^112^, ^113^, ^114^, ^115^

Emerging evidence suggests beta‐blockers may enhance cancer treatment efficacy by modulating apoptotic signaling pathways implicated in therapeutic resistance. Preclinical studies have shown beta‐blockers can promote apoptosis through diverse mechanisms in various malignancies. In gastric cancer models, the non‐selective beta‐blocker propranolol inhibited cell proliferation and induced apoptosis by downregulating anti‐apoptotic pathways like MEK–ERK.^116^ Propranolol similarly arrested cell cycle progression and triggered apoptosis in triple‐negative breast cancer cells, mediated by suppressing the ERK/COX‐2 pro‐survival pathway.^36^ Selective and non‐selective beta‐blockers also induced apoptosis in lung cancer and normal lung cells, although sensitivity varied based on the beta‐blocker subtype. The non‐selective agent nadolol most potently increased apoptotic signaling through mitochondrial and endoplasmic reticulum stress‐mediated mechanisms.^117^

Together, these findings indicate that beta‐blockers can modulate key apoptotic pathways to induce cancer cell death. However, effects appear dependent on beta‐blocker selectivity and cell context. Additionally, defects in apoptotic pathways often contribute to chemoresistance by impeding cell death.^118^, ^119^, ^120^ Additional research is needed to optimize beta‐blocker types and identify malignancies in which promoting apoptosis through these agents in combination regimens may improve therapeutic outcomes. Elucidating the signaling mechanisms involved will help guide translational efforts focused on apoptotic modulation (Table 3).

**TABLE 3:** Summary of preclinical and clinical studies on the impact of beta‐blockers on radiotherapy and immunotherapy.

Study type	Key findings	Reference
In vitro study (human gastric adenocarcinoma cell lines)	Propranolol sensitized cancer cells to radiation therapy by suppressing the NF‐κB‐VEGF/EGFR/COX‐2 pathway	[81]
In vitro and in vivo study (medulloblastoma)	Beta‐blockers improved radiotherapy efficacy by disrupting adaptive bioenergetic capacities, leading to enhanced DNA damage	[82]
In vitro study (adenocarcinoma cell lines)	Propranolol combined with radiation or cisplatin reduced cell survival and suppressed p‐PKA expression	[83]
Retrospective cohort study (non‐small‐cell lung cancer patients)	Beta‐blockers improved distant metastasis‐free survival and overall survival in patients receiving radiotherapy	[84]
Retrospective cohort study (low‐ to medium‐grade intracranial meningiomas)	Beta‐blockers improved outcomes in patients treated with stereotactic irradiation	[85]
Retrospective cohort study (esophageal cancer patients)	Concurrent use of beta blockers improved progression‐free survival and overall survival in patients receiving chemoradiation	[86]
Mouse model study (melanoma)	The combination of propranolol and immunotherapies delayed tumor growth and improved survival in mice	[87]
Retrospective analysis (metastatic melanoma patients)	Beta‐blockers improved overall survival in patients receiving immunotherapy	[87]
Retrospective study (non‐small cell lung cancer patients)	Beta‐blockers attenuated progression‐free survival in patients treated with immune checkpoint inhibitors	[88]
Phase III clinical trial (stage IIIA, IIIB, and IIIC melanoma patients)	Beta‐blockers may improve the efficiency of adjunct pembrolizumab treatment	[89]

## 8 CONCLUSIONS AND FUTURE DIRECTIONS

In summary, accumulating preclinical and clinical evidence indicates beta‐blockers may have broad utility in cancer therapy. Data supports their ability to inhibit cancer progression through anti‐angiogenic, anti‐metastatic, and anti‐proliferative effects. In addition, beta‐blockers show promise in reversing drug resistance to standard therapies like chemotherapy and enhancing treatment responses in diverse malignancies. Effects on the tumor microenvironment, cancer stem cell populations, and pro‐apoptotic signaling represent potential key mechanisms by which beta‐blockers modulate cancer progression and drug resistance. Moreover, our study represents recent evidence addressing the contribution of β3‐adrenoceptors in cancer progression, the role of antagonism of β3‐adrenoceptors in cancer treatment, and the positive impact of beta‐blockers on the responsiveness to radiotherapy and immunotherapy.

Larger randomized controlled trials are necessary to firmly establish the clinical efficacy and optimal utilization of beta‐blockers as adjuncts to existing cancer treatments. Key questions remain regarding the ideal beta‐blocker types, dosing schedules, and combination strategies with current therapies. Ongoing and future translational research studies will help address these knowledge gaps and determine whether beta‐blockers can be effectively incorporated into the anticancer armamentarium. Given their long history of safe use and low cost, beta‐blockers represent a promising pharmacological strategy against cancer progression and drug resistance if their clinical benefits are borne out.

## AUTHOR CONTRIBUTIONS

Simona Cavalu, Sameh Saber, Ahmed E. Amer, Rabab S. Hamad, Mustafa Ahmed Abdel‐Reheim, Elsayed A. Elmorsy, and Amir Mohamed Abdelhamid contributed to the conceptualization, literature review, editing, reviewing, interpretation, final edition, and approval of the manuscript.

## DISCLOSURES

The authors declare that they have no known competing financial interests or personal relationships that could have appeared to influence the work reported in this paper.

## Data Availability

Data sharing is not applicable to this article as no datasets were generated or analyzed during the current study.
